# Small-world properties of brain morphological characteristics in Anorexia Nervosa

**DOI:** 10.1371/journal.pone.0216154

**Published:** 2019-05-09

**Authors:** Enrico Collantoni, Paolo Meneguzzo, Elena Tenconi, Renzo Manara, Angela Favaro

**Affiliations:** 1 Department of Neurosciences, University of Padua, Padova, Italy; 2 Padua Neuroscience Center, University of Padua, Padova, Italy; 3 Radiology Unit, Department of Medicine and Surgery, Neuroscience section, University of Salerno, Salerno, Italy; University of Pennsylvania, UNITED STATES

## Abstract

Cortical thickness and gyrification abnormalities in anorexia nervosa (AN) have been recently described, but no attempt has been made to explore their organizational patterns to characterize the neurobiology of the disorder in the different stages of its course. The aim of this study was to explore cortical thickness and gyrification patterns by means of graph theory tools in 38 patients with AN, 20 fully recovered patients, and 38 healthy women (HC). All participants underwent high-resolution magnetic resonance imaging. Connectome properties were compared between: 1) AN patients and HC, 2) fully recovered patients and HC, 3) patients with a full remission at a 3-year follow-up assessment and patients who had not recovered. Small-worldness was greater in patients with acute AN in comparison to HC in both cortical thickness and gyrification networks. In the cortical thickness network, patients with AN also showed increased Local Efficiency, Modularity and Clustering coefficients, whereas integration measures were lower in the same group. Patients with a poor outcome showed higher segregation measures and lower small-worldness in the gyrification network, but no differences emerged for the cortical thickness network. For both cortical thickness and gyrification patterns, regional analyses revealed differences between patients with different outcomes. Different patterns between cortical thickness and gyrification networks are probably due to their peculiar developmental trajectories and sensitivity to environmental influences. The role of gyrification network alterations in predicting the outcome suggests a role of early maturational processes in the prognosis of AN.

## Introduction

Anorexia Nervosa (AN) is a disabling psychiatric disorder that typically develops in female individuals during adolescence or early adulthood and is characterized by important psychopathological, cognitive, medical and neurobiological abnormalities [1].

From a neurobiological perspective, in recent years many efforts have been made to describe brain volumetric and morphological characteristics in AN, and to characterize them according to the course of the disease and to different clinical variables. Since AN often has its onset in adolescence [2], a neurodevelopmental approach is of particular relevance in order to understand both the role of early etiopathogenetic factors [3] and the consequences of malnutrition on maturational trajectories.

The possibility to describe changes of morphological and structural brain features over neurodevelopmental trajectories allows better interpretation of how their alterations can impact on different psychiatric conditions.

The study of cortical thickness and gyrification indices are very promising within this context, since their modifications through the stages of brain maturation and their ability to capture anatomical and structural cortical properties are increasingly characterized [4]. During neurodevelopment, cortical thickness reflects processes that determine a progressive reorganization of grey matter structure, following the demands for greater plasticity in childhood and the subsequent need for higher synaptic stability in later phases. Gyrification, on the other hand, begins prior to birth to shape an efficient architecture that shows great structural stability over time, with the exception of a gradual decrease in the amount of cortical complexity during adolescence [5].

The different stability of these two structural parameters along neurodevelopmental trajectories is explained by their different sensitivity to environmental influences; cortical thickness is in fact more influenced by environmental exposures than gyrification, which maintains a more constant configuration during development [6,7].

Previous literature on brain morphology in patients with AN is inconsistent as regards findings, methodology approaches and recruitment criteria. A significant reduction of cortical thickness in underweight patients with AN was found by two studies, which did not detect a direct correlation between cortical thickness and body mass index (BMI) [8,9]. Furthermore, in a longitudinal study, Bernardoni and colleagues [10] observed a substantial normalization of thickness after an average of three months of weight restoration [10]. On the contrary, Lavagnino and colleagues [11], while observing a correlation between cortical thickness and BMI in the AN group, did not find any differences between patients and controls [11]. Moreover, in a recent study, a comparison between patients with AN (both acute and recovered) and healthy women revealed higher cortical thickness values in orbitofrontal areas [12].

Regarding cortical gyrification, Favaro et al. [13] observed the presence of significant alterations in the parietal and frontal cortex of adult patients with AN; hypogyrification in these areas was not correlated with weight loss, body mass index, cortical thickness or dehydration [13]. Furthermore, these alterations were not present in patients with a good clinical outcome, regardless of their body weight and recovery status. On the contrary, in a mixed sample of adolescents and adults, Bernardoni et al. [14] found that an almost complete normalization of cortical folding after weight gain and weight restoration was the main predictor of increased gyrification during treatment [14].

In the context of clinical neurosciences, evaluation of the morphological and structural parameters of the cerebral cortex on the basis of their covariance patterns is becoming increasingly important since it can reveal an inter-regional structural dependence, which derives from a complex mixture of developmental, genetic and environmental factors [15]. The possibility of characterizing the topology of cortical structural and morphological networks provides an insight into the ways in which the architecture of cortical connectivity negotiates the trade-off between network wiring cost and topological complexity and allows a very promising perspective on the study of psychiatric illnesses [16,17]. One of the most promising potentials of complex network sciences is in fact related to its applicability for uncovering developmental mechanisms that lead to aberrant brain network organization and for tracking the progression of disease in degenerative disorders [18]. The topological complexity of brain networks lies mainly in the need to mediate the presence of locally and globally distributed connections and to support, during neurodevelopment, the shift from a modular and segregated organization to a more globally integrated one. This integrated configuration fulfills the maturation of high order association areas and of higher cognitive abilities. In this perspective, it is of great interest to explore the connectomic characteristics of Anorexia Nervosa: a disorder that is hypothesized to have an early neurodevelopmental origin [19], but also represents a potential restraint for brain maturational trajectories due to starvation and consequent malnutrition. Our purpose in this paper is therefore to apply the tools provided by connectomics and graph theory to deepen our knowledge of the neurobiological complexity of AN, by trying to define any abnormalities in covariation patterns of gyrification and cortical thickness and then to assess the rules that govern the structural cortical topological interaction in the disorder. The secondary aim of the present study was to compare covariation patterns of the same brain characteristics between patients who have recovered at a 3-year follow-up and those who have not. We hypothesized that graph theory metrics in AN would support a delay in neurodevelopmental trajectories in both cortical measurements, with higher network segregation parameters and lower integrative properties in patients with AN compared to healthy controls (HC).

## Methods and materials

The sample included was the same as a previous study [13]. A total of 58 patients with AN (38 with acute AN and 20 fully recovered (AN-REC)) and 38 HC participated.

Patients with AN were recruited from the Padova Hospital Eating Disorders Unit. AN was defined according to DSM-5 criteria and all patients fulfilling the inclusion criteria who were in treatment or referred to the Unit while the study was being carried out were asked to participate. A sample of HC similar to the patient group in age, ethnicity, educational level, and hand lateralization was recruited from the same geographical area.

Exclusion criteria for both patients with AN and HC were male gender, history of head trauma or injury with loss of consciousness, history of any serious neurological or medical illness, active use of systemic steroids, pregnancy, active suicidality or major depression, history of substance/alcohol abuse or dependence, bipolar disorder or schizophrenia spectrum disorder, moderate mental impairment (IQ<60) or learning disabilities, use of medications other than antidepressants, and known contraindications to conventional MRI. History of any psychiatric disorder and any first-degree relatives with an eating disorder were additional exclusion criteria for HC.

When recruiting subjects, some individuals were not included in the study: five AN patients, because of antipsychotic medication and/or severe comorbidity; one AN patient and one healthy subject, because of previous head trauma; and one AN patient, 3 recovered AN and 2 healthy subjects, who were not available to undergo MRI scanning when scheduled. The final sample comprised of 96 women (38 with AN, 20 recovered from AN, and 38 HC). No further subject was excluded due to problems with scan acquisition, gross brain alterations, or motion artifacts.

The experimental sample was composed of different diagnostic subtypes: 32 subjects (84%) were restrictive AN, 6 patients were binge eating/purging AN subtype and 7 patients presented restrictive AN subtype with a history of binge eating or purging behavior. 14 AN patients and 4 recovered women were under drug treatment with antidepressants at the time of scanning (acute AN: 1 patient mirtazapine, 2 paroxetine, 2 escitalopram, 1 fluoxetine, 8 sertraline; recovered AN: 4 sertraline).

Definition of full recovery was: 1) having had AN (according to DSM-5 criteria) in their lifetime; 2) being asymptomatic for at least 6 months at the time of scanning (mean remission time: 38.5 months (standard deviation = 33.2; range 6–96). Amenorrhea, food restriction, bingeing, excessive exercise, fasting and purging in the last 6-months were exclusion criteria for the recovered AN group and none of the subjects of this group relapsed in the year following scanning. Table 1 describes the main characteristics of the sample.

Ethical permission was obtained from the ethics committee of the Hospital of Padova. After completely describing the study to the subjects, informed written informed consent was obtained.

**Table 1:** Baseline characteristics of the three groups.

	AN (n = 38)	AN-REC (n = 20)	HC (n = 38)	AN vs. HC	AN-REC vs. HC
	mean (SD)	mean (SD)	mean (SD)	z (p)	z (p)
Age (years)	26.1 (7.2)	26.3 (7.1)	25.3 (6.3)	0.38 (0.701)	0.44 (0.659)
Age at onset (years)	18.3 (5.1)	17.7 (3.2)	=	=	=
Duration of illness (months)	78.6 (81.3)	45.7 (65.0)	=	=	=
Duration of recovery (months)	=	45.4 (46.8)	=	=	=
Baseline BMI (kg/m^2^)	15.8 (1.8)	19.6 (1.6)	21.7 (2.9)	7.42 (0.000)	3.09 (0.002)
Lowest BMI (kg/m^2^)	14.0 (1.8)	15.7 (1.4)	19.8 (2.5)	7.17 (0.000)	5.35 (0.000)
Education (years)	14.2 (2.2)	14.2 (2.7)	15.5 (2.3)	2.63 (0.009)	1.94 (0.053)
Edinburgh laterality index	57.2 (37.6)	60.6 (35.2)	55.1 (42.0)	0.52 (0.603)	0.32 (0.749)
Left cortical thickness (mm)	2.45 (0.14)	2.52 (0.10)	2.53 (0.09)	2.65 (0.008)	0.23 (0.819)
Right cortical thickness (mm)	2.44 (0.14)	2.51 (0.11)	2.52 (0.08)	2.86 (0.004)	0.34 (0.731)
Left gyrification	2.85 (0.09)	2.90 (0.09)	2.90 (0.11)	1.97 (0.048)	0.23 (0.819)
Right gyrification	2.85 (0.10)	2.90 (0.09)	2.90 (0.12)	1.83 (0.067)	0.07 (0.941)

According to false discovery rate method, differences are significant at p<0.027

### Clinical assessment and follow-up

All subjects were investigated for AN diagnosis with a diagnostic interview according to the Eating Disorders Section of the Structured Clinical Interview for DSM-5 [20] and, also, a semi-structured interview was used in order to collect socio-demographic and clinical variables [21,22]. More information about subjects’ psychopathology was achieved using the Hopkins Symptoms Checklist [23] and the Eating Disorders Inventory [24] in order to gather depressive and obsessive-compulsive symptoms, as well as those regarding eating disorders. Furthermore, the Edinburgh Handedness Inventory [25] was used to assess handedness.

All subjects were recruited at the Hospital of Padova Eating Disorder Unit, fulfilled the diagnosis for AN according to DSM-IV criteria and were medically stable at the time of scanning. Most patients had restricting type anorexia nervosa at the time of scanning (see Supplementary Materials). Follow-up for acute AN patients was performed about 3 years later (average 3.4 years, range 1.7–3.9). A semi-structured interview, the Eating Disorders Section of the Structured Clinical Interview for DSM-IV, as well as information from informants, were used to achieve diagnostic information at follow up. Full recovery was defined as: normal range weight, regular menses, absence of binge/purge/avoidance or restrictive eating behavior, absence of excessive physical activity, body dissatisfaction or drive to thinness for at least 3 months before the evaluation. Table 2 shows the baseline characteristics of the two groups with a different outcome at follow-up.

**Table 2:** Baseline data of the two outcome groups.

	AN patients with recovery at follow-up (n = 13)	AN patients without recovery (n = 24)	z (p)
	mean (SD)	mean (SD)	
Age (years)	25.5 (6.8)	26.7 (7.5)	0.33 (0.74)
Age at onset (years)	20.8 (6.5)	17.1 (3.8)	2.26 (0.02)*
Duration of illness (months)	40.0 (46.2)	101.2 (89.8)	2.10 (0.04)
Baseline BMI (kg/m^2^)	14.9 (1.75)	16.2 (1.6)	2.23 (0.03)
Lowest BMI (kg/m^2^)	14.4 (2.0)	13.7 (1.7)	1.13 (0.26)
Duration follow-up (years)	3.2 (0.6)	3.5 (0.5)	1.48 (0.14)
Final BMI (kg/m^2^)	19.6 (2.1)	17.7 (4.3)	3.66 (<0.001)*

* According to false discovery rate method, differences are significant at p<0.027

### MRI data acquisition

Scans were collected using a Philips Achieva 1.5 Tesl scanner equipped for echo-planar imaging. High-resolution 3D T1-weighted anatomical images were acquired using a gradient-echo sequence (repetition-time = 20 sec, echo time = 3.78 msec, flip angle = 20°, 160 sagittal slices, acquisition voxel size = 1×0.66×0.66 mm, field of view 21–22 cm).

### Data processing and statistics

Data processing was performed using the FreeSurfer package (Martinos Center for Biomedical Imaging, Massachusetts General Hospital, Boston) version 5.3.0. Pre-processing, cortical reconstruction, segmentation, and cortical thickness estimation were performed according to standard protocols [26,27]—see Supplementary Materials for detailed information. Surface reconstruction and segmentation were manually inspected and minor manual intervention was performed when necessary, according to FreeSurfer user guidelines. The local Gyrification Index (lGI) was developed to take account of the three-dimensional nature of the cortical surface and was introduced in order to replace the previous two-dimensional linear gyrification measures, more susceptible to different kinds of bias. The lGI is a measure of cortical folding and was calculate at thousands of points of the reconstructed cortical surface using already validated algorithms [28]. The cortical surface was parcellated into 148 regions (74 in each hemisphere) using a specific suco-gyral atlas (Destrieux Atlas) (26).

### Constructing cortical thickness-based networks

A 148×148 Pearson’s correlation matrix of Cortical Thickness indices of each parcellated brain region was used to create a binary adjacency matrix for each group. The nodes of the matrix correspond to the areas parcellated according to the Destrieux atlas. Age, Edinburgh Handedness Inventory score and mean cortical thickness index were used as covariates.

A range of thresholds determined by connection densities (proportions of connections present in a graph to all possible connections) varying from 0.1 to 0.5 (increments of 0.05) was used to compare the properties of emerging networks.

### Constructing gyrification-based networks

A 148×148 Pearson’s correlation matrix of gyrification indices of each parcellated brain region was used to create a binary adjacency matrix for each group. The nodes of the matrix correspond to the areas parcellated according to the Destrieux atlas. Age, intracranial volume, Edinburgh Handedness Inventory score and mean overall gyrification index were used as covariates.

A range of thresholds determined by connection densities (proportions of connections present in a graph to all possible connections) varying from 0.1 to 0.5 (increments of 0.05) was used to compare the properties of emerging networks.

Comparing network measures between patients with different outcome profiles at follow up, the minimum density at which fully connected graph was observed was 0.20. Between these groups, the range of thresholds used to compare the properties of the networks varied from 0.2 and 0.5 (increments of 0.05).

### Properties of the connectome and group comparison

Covariance patterns within connectome are described using integration and segregation properties, which are quantified using various graph theory indices. Segregation indicates a modular development of related brain regions, while integration results from maturational processes affecting the entire brain. Integration was measured using Global Efficiency and Characteristic Path Length; segregation was measured using Clustering Coefficient, Modularity and Local Efficiency. We also quantified Small-World Index (SWI), a measure of the balance between integration and segregation. All topological properties were computed using Graph Analysis Toolbox (GAT) (https://www.nitrc.org/projects/gat/) [29].

Between group comparison was performed 2 groups at time, both for cortical thickness and for gyrification indices (AN vs HC, AN-rec vs HC, poor-outcome vs good-outcome). Significant differences between topological parameters were investigated using a nonparametric permutation test with 1000 repetition. The numerosity of the original groups were maintained in each repetition by the randomly reassignment of the regional data (or residuals) of each participant to one of the two group analyzed, so as to obtain an association matrix for each random group. Then, a range threshold of 0.1 to 0.5 with increments of 0.05 were applied to each random group in order to estimate the binary adjacency matrices. Topological measurements were calculated for all networks and the full density range were used to compare differences in network measurements. For each iteration, the values of each random group across the range of density were plotted and the differences of the different areas under the obtained curves were used to compute topological proprieties. p values were obtained by comparing the results from the actual differences in the curve functions obtained and the null distribution of differences. This nonparametric permutation test compared the shapes of the curves derived from multiple threshold points (and so from multiple comparisons) and is based on functional data analysis (FDA) that allowed to overcome limitations driven by the sensitivity of the analysis methodology. The same permutation procedure used to test the significance of the between group differences in global network measures are used to compare regional network measures.

### Graph-based metrics

Characteristic path length (CPL) is the number of edges that are present in the shortest path of two nodes, averaged over all pairs of nodes. High values of CPL indicate a less efficient flow of information across the connectome. Global efficiency (GE) is a measure of efficient information transfer and is inversely related to path length. Maximal GE values indicate a fully connected network.

The clustering coefficient indicates the density of connections between the neighbors of an individual node. The average of clustering coefficients across nodes indicate the clustering coefficient of the network. The local efficiency has a role similar to the clustering coefficient, representing a nodal measure of the average efficiency within a local subgraph. The modularity measures the correlation between the probability of having an edge that connect two nodes and the probability that nodes are part of the same community. Modules are defined as local communities of highly interconnected nodes which are poorly connected with other regions.

The small-world index (SWI) is computed by comparing the CPL and the CF of a graph with the corresponding values of null random graphs with same number of nodes, edges and degree distribution. Small world brain network exhibit the ability to use a relatively small number of long-distance connections to synchronize the information flow and the advantage to use local connections to locally processing information[30,31].

### Statistics

Group comparisons were performed by means of nonparametric statistical tests, with false discovery rate methods to control for multiple comparisons.

## Results

Table 1 shows the main clinical characteristics of the 3 groups involved in the study, including average cortical thickness and gyrification index as found in Favaro et al. [13]. Differences in vertex-wise analyses were reported in our previous paper [13].

### Cortical thickness based networks

Main findings regarding the properties of cortical thickness based networks are reported in Table 3. Hub distribution is described in Supplementary Materials.

**Table 3:** Topological properties of Cortical Thickness-based connectome.

	AN (n = 38)	HC (n = 38)	AN-REC (n = 20)	Poor outcome (n = 24)	Good outcome (n = 13)	FDA permutation test	
	Mean (SD)	Mean (SD)	Mean (SD)	Mean (SD)	Mean (SD)	(p-values)	Cohen’s d
Small-world index	1.64 (0.46)	1.45 (0.38)	1.63 (0.53)	1.66 (0.47)	1.73 (0.55)	AN>HC (0.0001)	0.45
***Measure of segregation***							
Clustering Coefficient	0.47 (0.08)	0.39 (0.11)	0.45 (0.09)	0.47 (0.08)	0.47 (0.78)	AN>HC (0.008)	0.83
Mean local efficiency	0.72 (0.07)	0.68 (0.09)	0.71 (0.07)	0.63 (0.09)	0.63 (0.09)	AN>HC (0.005)	0.50
Modularity	0.28 (0.09)	0.19 (0.07)	0.28 (0.09)	0.27 (0.08)	0.27 (0.09)	AN>HC (0.006)	1.12
***Measures of integration***							
Global efficiency	0.63 (0.09)	0.64 (0.08)	0.64 (0.09)	0.63 (0.09)	0.63 (0.09)	AN<HC (0.02)	0.12
Characteristic path length	1.78 (0.29)	1.74 (0.22)	1.77 (0.26)	1.78 (0.28)	1.79 (0.29)	AN>HC (0.03)	0.16

According to false discovery rate method, differences are significant at p<0.029

#### Patients with AN vs. HC

Patients with acute AN showed increased segregation measures in terms of Mean Local Efficiency, Clustering and Modularity in comparison to HC (Table 3), while, on the contrary, they revealed significantly lower patterns of integration as measured by Global Efficiency.

Both AN patients and HC reported average values of small-wordless greater than 1, but the small-world index was significantly higher in patients with acute AN than in HC (Table 3). No regional differences were detected either in the segregation or in the integration indices.

#### Patients recovered from AN vs. HC

No differences emerged in the comparison between the recovered AN and the healthy control group in any integration and segregation parameters in either overall or regional networks analysis. In recovered patients, mean small-world index was 1.63 (SD = 0.53). With regard to segregation measures, mean clustering coefficient was 0.45 (SD = 0.09), mean local efficiency is 0.71 (SD = 0.07) and mean modularity was 0.28 (SD = 0.09), whereas with regard to integration measures, mean global efficiency is 0.64 (SD = 0.09), and mean characteristic path length was 1.77 (SD = 0.26).

#### Good outcome patients vs. poor outcome patients

Patients with a poor outcome and those with a good outcome at a 3-year follow-up assessment did not show differences in global network properties. A regional analysis of the between-group differences revealed a significantly higher clustering coefficient of the orbital part of the left inferior frontal gyrus in the poor outcome group (FalseDiscoveryRate (FDR)-corrected permutation-based *p* values: <0.001) while patients with a good outcome show a higher degree in the same area (FDR-corrected permutation-based *p* values: <0.001).

### Gyrification based networks

Main findings regarding the properties of gyrification based networks are reported in Table 4. Hub distribution is described in Supplementary Materials.

**Table 4:** Topological properties of Gyrification-based connectome.

	AN (n = 38)	HC (n = 38)	AN-REC (n = 20)	Poor outcome (n = 24)	Good outcome (n = 13)	FDA permutation test	
	Mean (SD)	Mean (SD)	Mean (SD)	Mean (SD)	Mean (SD)	(p-values)	Cohen’s d
Small-world index	1.64 (0.46)	1.45 (0.38)	1.63 (0.53)	1.66 (0.47)	1.73 (0.55)	AN>HC (0.0001)	0.45
***Measure of segregation***							
Clustering Coefficient	0.47 (0.08)	0.39 (0.11)	0.45 (0.09)	0.47 (0.08)	0.47 (0.78)	AN>HC (0.008)	0.83
Mean local efficiency	0.72 (0.07)	0.68 (0.09)	0.71 (0.07)	0.63 (0.09)	0.63 (0.09)	AN>HC (0.005)	0.50
Modularity	0.28 (0.09)	0.19 (0.07)	0.28 (0.09)	0.27 (0.08)	0.27 (0.09)	AN>HC (0.006)	1.12
***Measures of integration***							
Global efficiency	0.63 (0.09)	0.64 (0.08)	0.64 (0.09)	0.63 (0.09)	0.63 (0.09)	AN<HC (0.02)	0.12
Characteristic path length	1.78 (0.29)	1.74 (0.22)	1.77 (0.26)	1.78 (0.28)	1.79 (0.29)	AN>HC (0.03)	0.16

According to false discovery rate method, differences are significant at p<0.029; n.s.: not significative

#### Patients with AN vs. HC

No differences were detected in integration and segregation measures between patients with AN and HC. Both AN patients, HC and recovered AN patients showed small-worldness greater than 1. However, the small-world index was significantly higher in patients with acute AN than in HC (Table 4).

#### Patients recovered from AN vs. HC

No statistically significant differences were detected in the gyrification based network in the comparison between recovered AN patients and HC. In recovered patients, mean small-world index was 1.81 (SD = 0.59). With regard to segregation measures, mean clustering coefficient was 0.54 (SD = 0.05), mean local efficiency was 0.76 (SD = 0.04) and mean modularity was 0.32 (SD = 0.11), whereas with regard to integration measures, mean global efficiency was 0.62 (SD = 0.10), and mean characteristic path length was 1.84 (SD = 0.36).

#### Good outcome patients vs. poor outcome patients

Patients with a poor outcome showed significantly higher clustering and trends towards significantly higher mean local efficiency and characteristic path length when compared to patients with a good outcome (Table 4). At a regional level, the poor outcome group revealed a higher normalized degree index in the inferior part of the right circular sulcus of the insula (FDR-corrected permutation-based *p* values: <0.001) and a higher normalized clustering of the left superior temporal sulcus (FDR-corrected permutation-based *p* values: <0.001).

Both groups showed small-worldness greater than 1, but the small-world index was significantly higher in the good outcome group. We performed the analysis including age at onset as a covariate and differences in small-world index remained statistically significant.

## Discussion

In the last decade many advances have been made in the description of the organizational principles that govern the anatomy and the topology of brain structural covariance networks and in establishing the relationship between them and functional connectivity patterns [15]. Regional and global structural brain features undergo profound modifications during development and establish their covariance properties following complex trajectories that are influenced by both genetic predisposition and environmental influences. The biological mechanisms underlying thickness and gyrification correlation among cortical areas might impact at different developmental stages and their properties should reflect the different mechanisms that influence the cortical connective architecture. Several studies have examined thickness and gyrification covariance patterns in psychiatric diseases in order to understand whether disruptions in segregation and integration properties are measurable and to investigate the candidate biological and developmental underpinnings that may explain such alterations [32–37].

In this study we use a connectomic approach by means of cortical thickness and gyrification data to study the cortical structural architecture in AN and to evaluate the presence of any imbalance in the overall cortical network properties and in regional subnetwork patterns.

Our results highlighted the presence of a significantly higher segregation of the overall cortical thickness network in the acute AN group in comparison to the healthy control group. AN patients in particular showed higher local efficiency, modularity and clustering coefficients, which indicate the presence of a more topologically localized and less densely distributed connective organization. On the contrary, gyrification patterns did not show significant differences between the acute AN and the HC group. The observation that cortical thickness networks showed more significant alterations than gyrification based networks in acute AN patients is probably due to their different developmental trajectory and to their different sensitivity to environmental changes. While convolution of the cortical surface begins prior to birth, establishing its main patterns principally in the fetal and neonatal stages, cortical thickness undergoes profound modification during later developmental phases, probably as the result of a fine tuning process between brain structure and function [38].

Evidence in the literature regarding the evolution of cortical thickness networks suggests that structural networks exhibit a global efficient small-world and modular organization by the time of birth and indicates a delayed increase of their integrative properties, which follows maturation of high order association areas and refinement of higher cognitive abilities [39,40].

In acute AN patients our results highlight the presence of significantly higher small-world properties in cortical thickness and in gyrification-based networks. These findings could represent the consequence of processes that tend to reduce the wiring cost of the global network and could indicate the presence of a more economical and less random cortical structural architecture. We can also hypothesize that this configuration could represent a consequence of the energy saving needs imposed by AN in the acute stage and that the presence of increased small-world properties is due to the attempt to maintain an adequate network efficiency despite starvation and malnutrition. This finding is also consistent with the clinical observation that many patients describe increased functioning during starvation, at least in the initial stages, and better ability in managing emotions, which is considered a maintenance factor [41].

The present study excludes the presence of any regional differences (which have been found in other psychiatric diagnoses) in integration and segregation properties, suggesting that the disorder impacts only the global covariation patterns estimated on cortical thickness indices. These results allow us to hypothesize that the acute effect of the disease could determine a global network readjustment that follows energy saving purposes. Therefore, AN could impact the balance between the wiring cost of the network and its integrative communication demand, supporting a less expensive connective architecture reconfiguration.

To better understand the impact of AN on the cortical structural connectivity architecture and on their developmental trajectories we evaluated a sample of AN recovered patients, without finding significant alterations either in gyrification or in cortical thickness covariance networks. These results seem to confirm the sensitivity of the overall cortical thickness network to the acute effects of the disease. In particular, we can hypothesize that nutritional status is crucial in determining the alterations in cortical thickness covariance patterns.

The second objective of our study was to explore the impact of gyrification and cortical thickness patterns in predicting outcome at follow up, evaluating the presence of possible differences in cortical connectivity patterns between “good prognosis” and “poor prognosis” patients. The absence of significant differences in the overall cortical thickness network properties between the two groups examined is consistent with a high cortical thickness sensitivity to the acute effects of AN, which homogeneously impact the cortical thickness covariance patterns in the acute stage of the disease.

However, regional analysis showed a non-homogeneous pattern, with a significantly lower number of connected edges (lower degree) and higher regional clustering of the orbital part of the left inferior frontal gyrus in the poor-outcome group. Inferior frontal gyrus is involved in numerous executive functions, playing a critical role mainly in cognitive control and in response inhibition. Proper maturation of inhibitory abilities is particularly crucial during adolescence, given the detrimental role of high levels of impulsivity during this critical developmental period. Interestingly, the transition from adolescence to adulthood is characterized by profound differences in the spatial localization of inhibitory processing, with a higher recruitment of right IFG in adulthood and a more left dominant processing at younger ages [42].

Adolescent individuals with AN show a bilateral decrease in gray matter volume in the IFG and the volume of this area was found to negatively correlate with both age and age of onset of the disorder [43]. Our observation of higher segregation in the IFG in the group of patients with a poor outcome is consistent with the idea that a lower integration of maturation of this area could mediate for prognostically unfavorable characteristics of the disorder.

Analysis of the role of gyrification-based networks in predicting the outcome at follow up showed the presence of a more clustered, segregated and less efficient overall covariance network in the poor-outcome group. Since gyrification patterns seem to develop mainly during the prenatal stages, our results are in line with others that support a neurodevelopmental hypothesis for AN and suggest a role of early maturational processes in the characterization of a subgroup of patients with low response to treatments.

Structural networks are proven to have an intimate relationship with functional cortical interconnectivity and a fundamental role in the way in which cortical regions structurally mature in relation to one another [44,45]. We can hypothesize that the higher global segregation of the poor-outcome group and its lower global efficiency could reduce the response to treatment by limiting the dynamic functional reconfiguration of the network and the information exchange between topologically distant brain areas. The finding of a higher small-world index in gyrification-based network in patients with a good outcome does not seem to contradict these hypothesis, since it probably reflects the presence of lower characteristic path length values in this group when compared to the good-outcome one.

The finding of a lower integration of gyrification indices in mediating the prognosis of AN is in line and expands the previous observation of a lower cortical gyrification in AN patients who show a poor outcome at a 3-year follow up [13]. In particular, since lower structural integration is likely to indicate a slowdown in the maturation of connectivity patterns during development, the opportunity to disentangle the role of neurodevelopment trajectories on the onset of AN and the impact that the disorder itself has on neurodevelopmental trajectories seems to be particularly relevant from a clinical point of view.

From a regional perspective, patients with a bad prognosis show an increased clustering of the left superior temporal sulcus, a higher-order processing region that has a key role in diverse aspects of social perception and cognition, including the perception of faces, voices and understanding the actions and mental states of others [46,47]. The higher clusterization of this area in AN patients who have a poor response to treatment indicates, in this clinical population, a preponderance of segregative network characteristics.

Patients with a poor outcome also have a reduced degree of the right insula, when compared with patients with good prognosis, which indicates a reduced centrality of an area with high integrative functions [48]. These regional differences are consistent with the high segregation of gyrification covariance networks in patients with bad prognosis and suggest a role of highly connected areas such as the insula and superior temporal sulcus in determining or mediating the resistance of the disorder to conventional treatments.

This study has several strengths, as well as important limitations, which should be taken into consideration. It is the first to analyze the relationships between different cortical structural indices using a connectomic framework in AN, and to describe the relationship between structural covariation patterns and subsequent outcomes of the disorder. However, particular caution must be applied in interpreting brain findings in AN samples, for which it is often difficult to disentangle the effects of early developmental factors on the brain and the consequences of starvation.

In conclusion, the present study highlights the presence of higher segregation and lower integration characteristics in the global cortical thickness-based network in patients with acute AN when compared to HC. These higher segregation characteristics could be due to a maturational delay, which would affect normal development trajectories, or to a protective and energy saving adaptation to the disease. However, the presence of small-world properties in AN patients guarantees the presence of non-random and balanced network properties, in line with the high level of functioning that characterizes patients with AN even in their malnourished status. The differences evidenced between cortical thickness and gyrification networks in acute AN patients and the observation of a more clustered and segregated gyrification network in patients with a bad prognosis suggest that the covariation patterns of these two parameters should be further investigated using longitudinal observations in order not only to better understand the long-term consequences of malnutrition, but also to explore the possibility of using gyrification and its pattern of covariance network as a measure of outcome.

## Supporting information

S1 File Methodological supporting information. Additional methodological information pertaining to the data utilized in this study.
(DOCX)
