# Supplementary material for: Small-world properties of brain morphological characteristics in Anorexia Nervosa
Source: PLoS One. 2019 May 9;14(5):e0216154. doi: 10.1371/journal.pone.0216154 (PMC6508864; doi:10.1371/journal.pone.0216154)
Supplement: S1 File — Additional methodological information pertaining to the data utilized in this study. (DOCX) [file pone.0216154.s001.docx]

**Supporting Information**

**Supplementary Methods**

Data Processing and Statistics

Surface extraction was completed using the FreeSurfer package (Martinos Center for Biomedical Imaging, Massachusetts General Hospital, Boston) version 5.3.0. The preprocessing was carried out according to the standard description using the following steps: skull-stripping and intensity correction, gray matter–white matter boundary determination for each cortical hemisphere using tissue intensity and neighborhood constraints, and finally, tessellation of the resulting surface boundary to generate multiple vertices across the whole brain before inflating.

After cortical reconstruction, the cortex was divided into units based on individual gyral and sulcal structures [1]. The local Gyrification Index (lGI) was measured at thousands of points of the reconstructed cortical surface using previously validated algorithms [2]. In each vertex, lGI is computed within 25-mm circular regions of interest and represents the degree of cortical folding that quantifies the amount of cortex buried with in the sulcal folds in the surrounding circular region. An overall hemispheric lGI value was automatically computed. Vertex-wise measurements of cortical thickness [3] were also estimated.

Graph-based metrics

Measures of integration, segregation and centrality were computed on each group-specific graph.

*Integration measures:*

1. path length is the minimum number of edges that must be traversed to go from one node to another. A measure of the typical separation between two nodes in the graph is given by the average shortest path length, also known as characteristic path length, defined as the mean of geodesic lengths over all couples of nodes [4].
2. Global efficiency is a measure of efficient information transfer and is inversely related to path lenght. A fully connected network has maximal global efficiency, while in a fully disconnected network the global efficiency has a minimal value.

*Segregation measures:*

1. The clustering coefficient of a node indicates the density of connections between the neighbors of the node. The clustering coefficient of a network is computed as the average of clustering coefficients across nodes.
2. Local efficiency has a role similar to the clustering coefficient, representing a nodal measure of the average efficiency within a local subgraph.
3. The modular organization of the connectome described by means of the Newman’s optimization algorithm [5] implemented in GAT. Modules are local communities of highly interconnected nodes which are poorly connected with other regions.

Centrality: We used two centrality measures to define the hubs in each group connectome: the degree of the nodes and the betweenness centrality.

1. The degree of a node is defined as the number of edges incident with the node.
2. The betweenness centrality of a node is computed on the number of shortest paths passing through it.

The small-world index (SWI) of a structural covariance network is computed by comparing the characteristic path length and clustering coefficient of a graph with the corresponding values of null random graphs with same number of nodes, edges and degree distribution. A SWI>1 indicates a network that has a relatively high segregation and integration compared to random null networks and suggests the presence of an efficient information transfer at a relatively low wiring cost [6].

Supplementary Results

We found differences in the distribution of the hubs based on degree and on betweenness centrality in the different groups. Hubs for each group are reported in Tables 1S, 2S, 3S, 4S, 5S, 6S

**Table 1S.** Hubs distribution in cortical thickness-based connectome. Comparison between AN patients and HC.

| **NET HUBS DEGREE**  **(AN)** | **NET HUBS DEGREE**  **(HC)** |
| --- | --- |
| L- Paracentral lobule and sulcus | L-Middle frontal sulcus |
| L- Middle frontal gyrus | L- Planum temporale or temporal plane of the superior temporal gyrus |
| L- Orbital sulci (H-shaped sulci) | L-Transverse temporal sulcus |
| R- Middle-anterior part of the cingulate gyrus and sulcus (aMCC) | R-Paracentral lobule and sulcus |
| R-Long insular gyrus and central sulcus of the insula | R-Short insular gyri |
| R- Postcentral gyrus | R- Postcentral gyrus |
| R-Superior frontal sulcus | R-Lateral aspect of the superior temporal gyrus |
|  | R- Temporal pole |
| **NET HUBS BETWENNESS**  **(AN)** | **NET HUBS BETWENNESS**  **(AN)** |
| L- Paracentral lobule and sulcus | L- Middle-anterior part of the cingulate gyrus and sulcus (aMCC) |
| L- Anterior part of the cingulate gyrus and sulcus(ACC) | L- Middle frontal gyrus (F2) |
| L- Posterior-dorsal part of the cingulate gyrus(dPCC) | L- Postcentral gyrus |
| L- Middle temporal gyrus (T2) | L- Planum polare of the superior temporal gyrus |
| R- Long insular gyrus and central sulcus of the insula | L- Superior occipital sulcus and transverse occipital sulcus |
| R- Middle occipital sulcus and lunatus sulcus | L- Transverse temporal sulcus |
| R- Anterior occipital sulcus and preoccipital notch (temporo-occipital incisure) | R- Short insular gyri |
| R- Fronto-marginal gyrus (of Wernicke) and sulcus | R- Postcentral gyrus |
|  | R- Lateral aspect of the superior temporal gyrus |

**Table 2S.** Hubs distribution in gyrification-based connectome. Comparison between AN patients and HC.

| **NET HUBS DEGREE**  **(AN)** | **NET HUBS DEGREE**  **(HC)** |
| --- | --- |
| L- Subcentral gyrus (central operculum) and sulci | L-Opercular part of the inferior frontal gyrus |
| L- Long insular gyrus and central sulcus of the insula | R- Opercular part of the inferior frontal gyrus |
| L- Lateral occupito-temporal gyris (fusiform gyrus) | R-Triangular part of the inferior frontal gyrus |
| R- Subcentral gyrus (central operculum) and sulci | R-Vertical ramus of the anterior segment of the lateral sulcus (or fissure) |
|  | R- Superior segment of the circular sulcus of the insula |
|  | R- Suborbital sulcus |
| **NET HUBS BETWENNESS**  **(AN)** | **NET HUBS BETWENNESS**  **(HC)** |
| L- Lateral occipito-temporal gyrus (fusiform gyrus, O4-T4) | L- Anterior part of the cingulate gyrus and sulcus(ACC) |
| L- Supramarginal gyrus | L- Inferior temporal gyrus (T3) |
| R- Superior occipital gyrus (O1) | L- Anterior transverse collateral sulcus |
| R- Orbital gyri | L- Posterior transverse collateral sulcus |
| R- Supramarginal gyrus | R- Middle-anterior part of the cingulate gyrus and sulcus (aMCC) |
| R- Anterior transverse collateral sulcus | R- Supramarginal gyrus |
| R- Lateral orbital sulcus | R- Intraparietal sulcus (interparietal sulcus) and transverse parietal sulci |
| R- Medial orbital sulcus (olfactory sulcus) | R- Inferior part of the precentral sulcus |
| R- Superior temporal sulcus (parallel sulcus) |  |

**Table 3S.** Hubs distribution in cortical thickness-based connectome. Comparison between AN-rec patients and HC.

| **NET HUBS DEGREE**  **(AN-rec)** | **NET HUBS DEGREE**  **(HC)** |
| --- | --- |
| L- Middle-posterior part of the cingulate gyrus and sulcus (pMCC) | L- Long insular gyrus and central sulcus of the insula |
| L- Straight gyrus, Gyrus rectus | L- Planum polare of the superior temporal gyrus |
| L- Marginal branch (or part) of the cingulate sulcus | L- Posterior ramus (or segment) of the lateral sulcus (or fissure) |
| R- Posterior-ventral part of the cingulate gyrus (vPCC, isthmus of the cingulate gyrus) | L- Superior temporal sulcus (parallel sulcus) |
| R- Anterior transverse temporal gyrus (of Heschl) | L- Transverse temporal sulcus |
| R- Superior occipital sulcus and transverse occipital sulcus | R- Superior parietal lobule (lateral part of P1) |
|  | R- Postcentral gyrus |
|  | R- Anterior transverse temporal gyrus (of Heschl) |
| **NET HUBS BETWENNESS**  **(AN)** | **NET HUBS BETWENNESS**  **(HC)** |
| L- Anterior part of the cingulate gyrus and sulcus (ACC) | L- Long insular gyrus and central sulcus of the insula |
| L- Posterior-ventral part of the cingulate gyrus (vPCC, isthmus of the cingulate gyrus) | L- Postcentral gyrus |
| L- Calcarine sulcus | L- Planum polare of the superior temporal gyrus |
| L- Marginal branch (or part) of the cingulate sulcus | L- Posterior ramus (or segment) of the lateral sulcus (or fissure) |
| R- Cuneus | L- Superior temporal sulcus (parallel sulcus) |
| R- Posterior transverse collateral sulcus | L- Transverse temporal sulcus |
| R- Middle frontal sulcus | R- Superior parietal lobule (lateral part of P1) |
| R- Pericallosal sulcus (S of corpus callosum) | R- Anterior transverse temporal gyrus (of Heschl) |

**Table 4S.** Hubs distribution in gyrirification-based connectome. Comparison between AN-rec patients and HC.

| **NET HUBS DEGREE**  **(AN-rec)** | **NET HUBS DEGREE**  **(CTR)** |
| --- | --- |
| L- Middle-anterior part of the *cingulate gyrus and sulcus* (aMCC) | L- *Opercular part of the inferior frontal gyrus* |
| L- *Calcarine sulcus* | R- *Triangular part of the inferior frontal gyrus* |
| R- Middle-anterior part of the *cingulate gyrus and sulcus* (aMCC) | R- Medial orbital sulcus (*olfactory sulcus*) |
| R- *Straight gyrus*, Gyrus rectus |  |
| R- Medial orbital sulcus (*olfactory sulcus*) |  |
| **NET HUBS BETWENNESS**  **(AN)** | **NET HUBS BETWENNESS**  **(AN)** |
| L- Precuneus (medial part of P1) | L- Anterior part of the cingulate gyrus and sulcus (ACC) |
| L-Short insular gyri | L- Planum temporale or temporal plane of the superior temporal gyrus |
| L- Vertical ramus of the anterior segment of the lateral sulcus (or fissure) | L- Postcentral sulcus |
| R- Long insular gyrus and central sulcus of the insula | R- Middle-anterior part of the cingulate gyrus and sulcus (aMCC) |
| R- Occipital pole | R- Lingual gyrus, ligual part of the medial occipito-temporal gyrus, (O5) |
| R- Calcarine sulcus | R- Orbital gyri |
|  | R- Intraparietal sulcus (interparietal sulcus) and transverse parietal sulci |
|  | R- Superior occipital sulcus and transverse occipital sulcus |

**Table 5S.** Hubs distribution in cortical thickness-based connectome. Comparison between patients with a good outcome and patients with a poor outcome.

| **NET HUBS DEGREE**  **(good-outcome group)** | **NET HUBS DEGREE**  **(poor-outcome group)** |
| --- | --- |
| L- Middle frontal sulcus | L- Middle-anterior part of the cingulate gyrus and sulcus (aMCC) |
| L- Inferior part of the precentral sulcus | L- Superior parietal lobule (lateral part of P1) |
| R- Angular gyrus | L- Intraparietal sulcus (interparietal sulcus) and transverse parietal sulci |
| R- Middle frontal gyrus (F2) | R- Postcentral gyrus |
| R- Sulcus intermedius primus (of Jensen) | R- Superior frontal sulcus |
|  | R- Anterior occipital sulcus and preoccipital notch(temporo-occipital incisure) |
| **NET HUBS BETWENNESS**  **(good-outcome group)** | **NET HUBS BETWENNESS**  **(poor-outcome group)** |
| L-Middle-anterior part of the cingulate gyrus and sulcus (aMCC) | L- Posterior-dorsal part of the cingulate gyrus(dPCC) |
| L- Anterior transverse temporal gyrus (of Heschl) | L- Inferior segment of the circular sulcus of the insula |
| L- Occipital pole | L- Superior segment of the circular sulcus of the insula |
| L- Temporal pole | L- Sulcus intermedius primus (of Jensen) |
| L- Middle frontal gyrus (F2) | R- Occipital pole |
| L- Lateral orbital sulcus | R- Superior frontal gyrus (F1) |
| L- Parieto-occipital sulcus (or fissure) | R- Middle occipital sulcus and lunatus sulcus |
| R- Inferior segment of the circular sulcus of the insula | R- Anterior occipital sulcus and preoccipital notch(temporo-occipital incisure) |
| R- Medial orbital sulcus (olfactory sulcus) |  |

**Table 6S.** Hubs distribution in gyrification-based connectome. Comparison between patients with a good outcome and patients with a poor outcome.

| **NET HUBS DEGREE**  **(good-outcome group)** | **NET HUBS DEGREE**  **(poor-outcome group)** |
| --- | --- |
| L- Opercular part of the inferior frontal gyrus | L- Anterior transverse temporal gyrus (of Heschl) |
| L- Lateral aspect of the superior temporal gyrus | R- Opercular part of the inferior frontal gyrus |
| R- Lateral orbital sulcus |  |
| **NET HUBS BETWENNESS**  **(good-outcome group)** | **NET HUBS BETWENNESS**  **(poor-outcome group)** |
| L- Opercular part of the inferior frontal gyrus | L- Posterior ramus (or segment) of the lateral sulcus (or fissure) |
| L- Lateral aspect of the superior temporal gyrus | L- Marginal branch (or part) of the cingulate sulcus |
| L- Inferior frontal sulcus | L- Subparietal sulcus |
| L- Superior temporal sulcus (parallel sulcus) | R- Anterior part of the cingulate gyrus and sulcus(ACC) |
| R- Middle-posterior part of the cingulate gyrus and sulcus (pMCC) | R- Opercular part of the inferior frontal gyrus |
| R- Opercular part of the inferior frontal gyrus | R- Lateral occipito-temporal gyrus (fusiform gyrus, O4-T4) |
| R- Subcallosal area, subcallosal gyrus | R- Pericallosal sulcus (S of corpus callosum) |
| R- Lateral orbital sulcus |  |

REFERENCES:

1. Destrieux C, Fischl B, Dale A, Halgren E. Automatic parcellation of human cortical gyri and sulci using standard anatomical nomenclature. Neuroimage. NIH Public Access; 2010;53: 1–15. doi:10.1016/j.neuroimage.2010.06.010

2. Schaer M, Bach Cuadra M, Tamarit L, Lazeyras F, Eliez S, Thiran JP. A Surface-based approach to quantify local cortical gyrification. IEEE Trans Med Imaging. 2008;27: 161–170. doi:10.1109/TMI.2007.903576

3. Fischl B, Dale AM. Measuring the thickness of the human cerebral cortex from magnetic resonance images. Proc Natl Acad Sci. 2000;97: 11050–11055. doi:10.1073/pnas.200033797

4. Boccaletti S, Latora V, Moreno Y, Chavez M, Hwang D-U. Complex networks: Structure and dynamics. Phys Rep. 2006;424: 175–308. doi:10.1016/j.physrep.2005.10.009

5. Newman MEJ, Girvan M. Finding and evaluating community structure in networks. Phys Rev E. 2004;69: 26113. doi:10.1103/PhysRevE.69.026113

6. Rubinov M, Sporns O. NeuroImage Complex network measures of brain connectivity : Uses and interpretations. Neuroimage. Elsevier Inc.; 2010;52: 1059–1069. doi:10.1016/j.neuroimage.2009.10.003
